# Long-term retention assessment after simulation-based-training of pediatric procedural skills among adult emergency physicians: a multicenter observational study

**DOI:** 10.1186/s12909-019-1793-6

**Published:** 2019-09-11

**Authors:** Raihei Ansquer, Thomas Mesnier, Farnam Farampour, Denis Oriot, Daniel Aiham Ghazali

**Affiliations:** 1 Emergency Department and EMS, University Hospital of Poitiers, 86000 Poitiers, France; 2 Emergency Department and EMS, General Hospital of Angouleme, 16000 Angouleme, France; 3 Emergency Department and EMS, General Hospital of Niort, Niort, France; 4 Pediatric Emergency Department, University Hospital of Poitiers, 86000 Poitiers, France; 5 ABS Lab - Simulation Laboratory, Faculty of Medicine, University of Poitiers, Poitiers, France; 6 Emergency Department and EMS, University Hospital of Bichat and Beaujon, Paris, France; 7 Ilumens- Simulation Laboratory, Faculty of Medicine, University of Paris-Diderot, 75018 Paris, France; 8 Simulation center, University Paris-Diderot, 20 rue du Département, 75018 Paris, France; 9 Emergency Medical Service and Emergency Department, University Hospital of Bichat and Beaujon, 46 rue Huchard, 75018 Paris, France

**Keywords:** Training, Education, Long-term memory, Simulation-based education, Performance, Emergency medicine, Technical skills, Pediatric emergency, Assessment

## Abstract

**Background:**

One of the primary goals of simulation-based education is to enable long-term retention of training gains. However, medical literature has poorly contributed to understanding the best timing for repetition of simulation sessions. There is heterogeneity in re-training recommendations.

**Objectives:**

This study assessed, through simulation-based training in different groups, the long-term retention of rare pediatric technical procedures.

**Methods:**

This multicenter observational study included 107 emergency physicians and residents. Eighty-eight were divided into four groups that were specifically trained for pediatric emergency procedures at different points in time between 2010 and 2015 (< 0.5 year prior for G1, between 0.5 and 2 years prior for G2, between 2 and 4 years prior for G3, and ≥ 4 years prior for G4). An untrained control group (C) included 19 emergency physicians. Participants were asked to manage an unconscious infant using a low-fidelity mannequin. Assessment was based on the performance at 6 specific tasks corresponding to airway (A) and ventilation (B) skills. The performance (scored on 100) was evaluated by the TAPAS scale (Team Average Performance Assessment Scale). Correlation between performance and clinical level of experience was studied.

**Results:**

There was a significant difference in performance between groups (*p* < 0.0001). For G1, 89% of the expected tasks were completed but resulted in longer delays before initiating actions than for the other groups. There was no difference between G4 and C with less than half of the tasks performed (47 and 43% respectively, *p* = 0.57). There was no correlation between clinical level of experience and performance (*p* = 0.39).

**Conclusion:**

Performance decreased at 6 months after specific training for pediatric emergency skills, with total loss at 4 years after training, irrespective of experience. Repetition of simulation sessions should be implemented frequently after training to improve long-term retention and the optimal rate of refresher courses requires further research.

## Introduction

One of the primary goals of education is to enable the retention of long-term gains in knowledge and/or skill [1]. In medical education, physicians are trained to transfer and adapt their knowledge to many different future clinical challenges, based on a retained skillset. In France, emergency physicians’ education includes only a very small part devoted to pediatric emergency medicine. Moreover, the incidence of critical illness, particularly cardio-pulmonary arrest and injury in children, is much lower than in adults [2]. Consequently, providers with limited knowledge and experience manage most pediatric emergencies. This explains why pediatric emergency care can be considered as an infrequent and complex practice for emergency providers. Rarely practiced skills can be a source of failure or complication [3]. Furthermore, unpredictable situations for emergency teams may develop stress that can lead to poor management of life-threatening events [4]. Simulation-based education (SBE) enhances skills in pediatric emergencies such as neonatal resuscitation [5], pediatric advanced life support, and procedural training [6, 7]. To date, several studies have demonstrated the value of integrating simulation into the medical curriculum to improve knowledge, skills, and behaviors [8–11]. Simulation training would improve decision-making and procedural skills for rare and critical events [12, 13]. In 2012, the French National Authority for Health (HAS) concluded that simulation could reduce the gap between low exposure to critical situations and the repeated practice necessary for efficient management [14]. However, the impact of memory retention after training for complex technical skills is difficult to assess [1]. In 2013, a meta-analysis on simulation and pediatric teaching recognized the lack of educational simulation patterns necessary to acquire and maintain skills because of scarcity of comparative studies in medicine [15]. Despite the increase of publications on SBE in pediatrics, many of these new studies use a “no intervention group” as a control and poorly contribute to understand what is the best delay before repetition of simulation sessions [15]. Moreover, among the pediatric simulation trainings, there is heterogeneity in re-training recommendations [16–18]. Therefore, it is of interest to assess long-term retention of the skills acquired after simulation training and to determine factors that can influence this pedagogical process. The assumption is that technical performance increases after a simulation-based training session [15, 19–21] and decreases more or less rapidly afterwards until the return to baseline knowledge.

The goal of this study was to assess essential but rarely used technical skills in pediatric emergencies among groups of emergency physicians who had received relevant simulation-based training at different time points prior to the assessment. It also aimed to identify the potential influence of stress and experience on this retention process.

## Methods

### Design

This multicenter observational study was a cross-sectional study of skill retention in different groups including residents and physicians in Emergency Medicine. It took place in the Emergency Departments of the French Grande Aquitaine region (four medical centers) and in the hospital of Cayenne (French Guyana) between April and July 2015. Assessment of performances was based on the European Resuscitation Council recommendations of 2010 before their modification in 2015. Assessment of performance was performed in the simulation center of the University of Poitiers (Poitiers, France) and in the simulation center of the University of Paris-Diderot (Paris, France).

### Objectives

The primary objective of this study was to assess the impact of time passed since SBE training on retention of medical technical skills relevant to pediatric emergency scenarios.

Secondary objectives were: 1) to compare completion of required skills; 2) to measure the time to complete tasks; 3) to assess participants’ feelings; 4) to analyze the link between performance, perceived stress, and clinical level of experience.

### Population

Participation in this research was on a voluntary basis. Firstly, emergency residents and emergency physicians, carrying out the university course of Pediatrics Emergency Procedures (PEP) between 2010 and 2015, were requested to participate by e-mail. Only participants living in the Grande Aquitaine region and in French Guyana were contacted. Secondly, emergency physicians of the region who had not yet taken the PEP course were requested by e-mail to participate in the control group (C). Non-inclusion criteria were: 1- Pediatricians or emergency physicians working in a pediatric emergency department; 2- Participants who had not completed or not validated the university course (i.e. having scored less than 14/20, except for the control group); 3- Participants who had been given other pediatric training; 4- Participants who did not give consent for video were not included. Participants who filled out the survey incompletely were also excluded.

Four groups (G1 to G4) were formed based on the dates of their PEP course final exam: G1 had completed training less than 0.5 year before; G2 between 0.5 and 2 years before; G3 between 2 and 4 years before; and for G4, at least 4 years before. A fifth group was the control group (C).

### Rationale for the chosen period

The choice for G1 was based on the literature on ventilation assessment [22, 23]. For G2 it was based on the recommendations for retraining of health personnel every 2 years, for example according to the Resuscitation Council Worldwide and the American Heart Association recommendations [17, 18]. For G3 and G4, it was based on recommendations to recycle at least every 4 years, as in Advanced Trauma Life Support (ATLS) [24].

### Intervention

Prior to the specific scenario testing, a prerequisite training in pediatric emergency was mandatory for groups. Between 2010 and 2015, all participants except for the control group (C), had completed an identical pediatrics emergency training program called the PEP university course (based on the most recent European Resuscitation Council recommendations). A testing scenario was drawn from among a series of three scenarios concerning ventilation, circulation, or neurological life-threatening events. The participants had to manage an unconscious three-month-old infant during a videotaped and timed low-fidelity simulation. Information about the PEP university course and the scenario is given in Additional file 1.

All simulations were standardized, including briefing (5mn), simulation (5mn maximum to achieve all requested objectives to manage the life-threatening event), and debriefing (20mn). During the briefing, it was indicated to participants that they were the first witnesses of a life-threatening emergency and that they had to manage the first few minutes prior to the arrival of other caregivers. The situation was described in a similar way to all participants. A standardized debriefing with good judgment method was carried out [25].

Raters were supervisors in the PEP course with more than 5 years of experience in simulation and in rating of technical performance according to the TAPAS scale (Team Average Performance Assessment Scale) [26]. Raters were trained to use this scale by its developers. All simulations were assessed by two raters. The simulation was videotaped to reduce assessment bias and to allow accurate analysis of the performance. The video was displayed in case of uncertainty of ranking or discordance between the raters’ assessments.

### Assessment tools

Assessment tools used objective and subjective evaluations.

#### Assessment of technical performance

Technical procedure performance and time assessment were carried out using the TAPAS scale, that we previously designed and validated (Cronbach α = 0.745; Intra-Class Coefficient = 0.862) [26]. The TAPAS scale is based on international recommendations and evaluates the technical skills applied during the ABCDE approach. Each item is rated 0, 1 or 2 (0: not performed, 1: performed too late or poorly, 2 in time and correctly performed). The expected items are given in Additional file 2. Items for the airway (A) sequence were: responsiveness checking, putting the child in neutral position, airway opening, inspecting the mouth and suctioning of secretions, inserting an oral airway. The expected items for breathing (B) sequence were: checking of respiratory rate, performing Bag-Valve-Mask (BVM) ventilation with 5 initial breaths during 3 s followed by a ventilation rate of 25–30 /min. At the end of the sequence the participants had to check brachial pulse. A gastric tube had to be inserted before the end of the sequence. The average of the scores calculated by the two raters was considered as the participant’s performance score. In addition to performance score, the percentage of items performed in each group was given. The time was measured in seconds for completion of the 6 main items: search of responsiveness (T1), neutral positioning (T2), insertion of oral airway (T3), beginning of BVM ventilation (T4), pulse checking (T5), and insertion of gastric tube (T6).

#### Assessment of self-reported level of stress, confidence, dissatisfaction and perceived realism

Participants’ perceived stress was assessed by the Stress-O-Meter (SOM) scale with score of 0 (None) to 10 (Maximal) at the beginning of the standardized debriefing [27, 28].

During the first phase of debriefing, we also assessed perceived self-confidence, feeling of dissatisfaction, and realism of the simulation using a 0–10 Likert scale.

#### Questionnaire

An anonymous questionnaire (Additional file 3) was used to collect information on the characteristics of the participants. The participants’ experience level in emergency medicine was noted in years.

### Statistical analysis

All data were de-identified and analyzed with Excel 2013 software, and Statview Version 4 .5 (SAS Institute Inc., Cary, NC). To facilitate statistical interpretation, scores and results of questionnaire were reported on a 100-scale with the proportionality rule. The Shapiro-Wilk test was used to evaluate the normal distribution. Ordinal and continuous variables were expressed by mean and standard deviation or by median and [1st, 3rd quartile]. Data were analyzed with a series of pair-wise comparisons using the Mann-Whitney U test, and the overall main effect of groups with the Kruskal-Wallis nonparametric test. The categorical variables were expressed by number and percentage (%) and were compared by a Chi^2^ test. A link between performance and experience was investigated by Spearman’s linear correlation coefficient. A *p* value < 0.05 was considered significant.

### Ethics

This study was considered as an evaluation of the professional practices by the French National Safety Agency. The Simulation Laboratory of the Faculty of Medicine and Pharmacy at Poitiers University was accredited by the Regional Health Agency of Poitou-Charentes for biomedical research on healthy volunteers (January 28th, 2013). The Research Board and local ethics committee of the Faculty of Medicine of Poitiers approved this research. Participants were informed and written consent was obtained for the participation and the use of the video. Results were kept de-identified.

## Results

### Population

Two hundred and sixty-two participants attended the PEP course between 2010 and 2015. One hundred and thirty-seven participants were recruited. Thirty participants were not included: 5 pediatricians, 18 emergency physicians who had encountered other pediatric simulations, 5 emergency physicians who did not validate the PEP course, and 2 emergency physicians who did not consent to the video recording. Finally, 107 participants were included in the study and were divided into five groups (G1 to G4 and C). G1 had 23 participants, G2 had 20, G3 had 22, G4 had 23, and the control group (C) had 19 participants. The baseline characteristics of the participants are summarized in Table 1.

**Table 1:** Characteristics of the participants

Groups	G1	G2	G3	G4	Control
Number of participants (n, %)	23 (21.5)	20 (18.7)	22 (20.6)	23 (21.5)	19 (17.7)
Male (n, %)	13 (56.5)	11 (55.0)	12 (54.5)	13 (56.5)	11 (57.9)
Physicians	18	16	22	23	19
Residents	5	4	0	0	0
Age (M ± SD)	31 ± 3	32 ± 3	34 ± 1	35 ± 2	32 ± 3
Years of clinical experience (median[Q1.Q3])	7[2;8]	2[1;2]	2[2;3]	15[7;17]	3[3;9]

Legend: Groups: G1 had completed training < 0.5 year ago; G2 between 0.5 and 2 years ago; G3 between 2and 4 years ago, G4 ≥ 4 years ago, Control: untrained group

*M* mean, *SD* standard deviation, *Q1* first quartile, *Q3* third quartile

### Main outcome

**Fig. 1:**
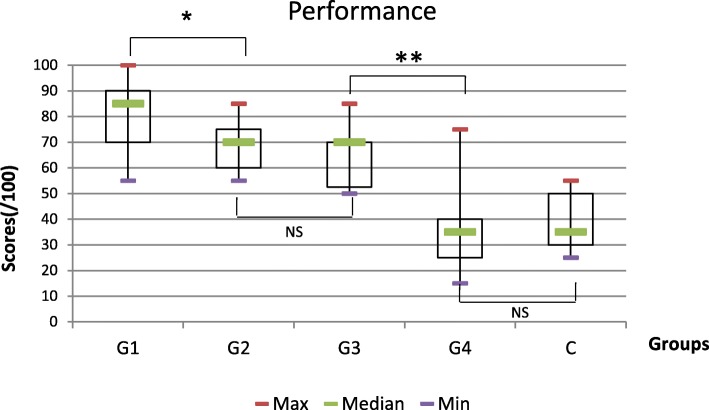
Box Plots for overall performance scores for management of an unconscious infant in each group (Medians, first and third quartiles). Legend: NS = not significant; *p < 0.01; **p < 0.001. Groups: G1 had completed training < 0.5 year ago; G2 between 0.5 and 2 years ago; G3between 2and 4 years ago, G4 *≥* 4 years ago, Control: untrained group

Performance scores significantly decreased over time (*p* < 0.0001) (Fig. 1). The highest scores were measured in G1: 85 [80; 90]. Performance scores significantly decreased after 6 months (*p* = 0.002). Scores were 70 [60; 75] in G2 and 70 [53; 70] in G3 respectively meaning a 17.4% performance reduction. Then, performance scores significantly decreased after 4 years (*p* < 0.001). In G4, an additional 50% drop in performance when compared to G3 was found with a score of 35 [25; 40]. Identical scores of 35 [30; 50] were measured in the control group compared to G4 (*p* = 0.70). Pair-wise comparisons of the performance score two by two are given in Table 2.

**Table 2:** Comparison of performance scores between the groups for management of an unconscious infant

Groups	G2	G3	G4	Control
G1	***p*** = 0.002*	***P*** < 0.001*	***p*** < 0.001*	***p*** < 0.001*
G2	NA	*P* = 0.48	***p*** < 0.001*	***p*** < 0.001*
G3	NA	NA	***p*** < 0.001*	***p*** < 0.001*
G4	NA	NA	NA	*P* = 0.70

Legend: Groups: G1 had completed training < 0.5 year ago; G2 between 0.5 year and 2 years ago; G3 between 2 and 4 years ago, G4 ≥ 4 years ago, Control: untrained group

*NA* Not applicable

*p < 0.05

### Secondary outcomes

#### Comparison of procedural steps performed in each group

**Fig. 2:**
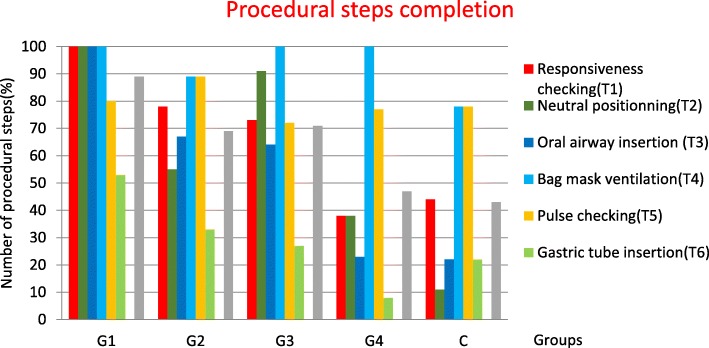
Procedural steps performed in each group during management of an unconscious infant. Legend: Groups: G1 had completed training < 0.5 year ago; G2 between 0.5 and 2 years ago; G3 between 2 and 4 years ago, G4 ≥ 4 years ago, Control: untrained group

The responsiveness assessment (T1), neutral positioning (T2), and insertion of oral airway (T3) were performed by all participants of G1 (Fig. 2). These procedural steps were performed by over 50% of the participants of G2 and G3. Less than 50% of participants of G4 and control group (C) performed these procedures. In all groups, more than 70% of participants carried out BVM ventilation (T4) and pulse checking (T5). The gastric tube was inserted by more than 50% of the G1 participants and less than 33% of those of G2. Overall, the G1 participants performed 89% of the procedural steps that were expected in the scenario. In contrast, the G4 and C participants performed less than 50% of them.

BVM ventilation (T4) and pulse checking (T5) were the most frequently performed procedural steps for the entire cohort (93 and 79%, respectively). Gastric tube insertion (T6) was the least performed step, only 29% of the time.

#### Analysis of the time for completion for each procedural step in the management of the case

There was a significant difference between groups for T4 and T5 (Table 3). G1 performed BVM ventilation (T4) and pulse checking (T5) with longer durations than other groups. T4 was performed in 93 s [87; 108] and T5 was performed in 122 s [104; 161] for G1. Control group participants checked the pulse (T5) faster than in any other groups with a median of 16 s [13; 26].

**Table 3:** Comparison between the different groups of median [1st; 3^rd^quartile] times (in seconds) to perform each procedural step during management of an unconscious infant

Groups	G1	G2	G3	G4	Control	*p*
Steps						
Responsiveness checking (T1)	2[2;5]	0[0;2]	3[2;6]	13[3;18]	9[2;15]	0.16
Neutral Positioning (T2)	19[16;24]	26[16;28]	14[7;27]	77[29;89]	80[80]	0.15
Oral airway insertion (T3)	55[48;65]	49[40;57]	37[32;68]	27[25;78]	112[82;142]	0.50
Bag mask ventilation (T4)	93[87;108]	48[37;64]	58[39;63]	41[30;45]	61[35;66]	0.001*
Pulse checking (T5)	122[104;161]	40[25;47]	80[54;96]	46[23;72]	16[13;26]	< 0.001*
Gastric tube insertion (T6)	126[77;155]	123[122;139]	133[115;137]	239[239]	138[116;161]	0.98

Legend: Groups: G1 had completed training < 0.5 year ago; G2 between 0.5 and 2 years ago; G3 between 2 and 4 years ago, G4 ≥ 4 years ago, Control: untrained group

*p < 0.05

#### Analysis of participant perceptions

There was no significant difference between groups for the perceptions of self-confidence (*p* = 0.15), pre-simulation stress (*p* = 0.12), feeling of dissatisfaction (*p* = 0.47), and realism of the scenario (*p* = 0.14). Level of perceived stress during simulation was higher in G1 (score of 8/10, *p* = 0.02) than the other groups (Table 4).

**Table 4:** Comparison of the perceptions scores on 0–10 scales (medians [1st; 3rd quartile])

Perceptions/Groups	G1	G2	G3	G4	Control	P
Self-confidence	5[4;6]	6[5;7]	6[4;7]	6[5;7]	4[1;5]	0.15
Pre-simulation Stress	6[5;8]	3[2;6]	3[2;5]	3[2;5]	4[2;5]	0.12
Stress during simulation	8[7;8]	5.5[5;6]	6[6;7]	5[3;6]	6.5[5;8]	0.02*
Feeling of dissatisfaction	5[5;6]	6[6;8]	7[5;7]	5[4;7]	6[5;9]	0.47
Realism of the scenario	8[7;9]	7[6;7]	5[4;6]	7[3;9]	5[3;8]	0.14

Legend: Groups: G1 had completed training < 0.5 year ago; G2 between 0.5 and 2 years ago; G3 between 2 and 4 years ago, G4 ≥ 4 years ago, Control: untrained group

*p < 0.05

#### Correlation between performance, clinical experience and perceived stress

Among participants, there was no correlation between clinical level of experience and performance score (*p* = 0.39). There was no correlation between perceived stress during simulation and clinical level of experience (*p* = 0.19). Similarly, there was no correlation between perceived stress during simulation and performance score (*p* = 0.13).

## Discussion

### Main results

While the medical technical performance of rare pediatric procedures was high just after simulation training (G1), it rapidly declined by 15% after 0.5 year (G2). This performance remained on a plateau until less than 4 years (G3). Then it decreased again by 35% until it was completely lost at 4 years (G4), descending to a level identical to absence of training (as in the control group) (C). To our knowledge there have been no studies in the literature studying skill retention beyond a 2 year delay, for technical skills pertaining to rarely performed procedures [26, 29, 30]. The group that had just completed the training (G1) had performed BVM ventilation and pulse checking with longer durations, while the group without training (C) had checked pulse faster than any other group. Immediately after training (G1), simulated case management was carried out more completely with a longer delay compared to the other groups. Over time, the specific pediatric procedural steps were less and less often carried out until their frequency became identical to the one of the untrained group (C). Among the different perception categories, the only difference found was that perceived stress during simulation was higher for G1 than in the other groups. There was no correlation between stress, performance, and clinical level of experience.

#### Primary outcome

This study demonstrated that technical performance after a simulation-based training was maximal within the 6 months following the training and followed by a drop as previously suggested in the literature [19, 21]. All participants had no other training or simulation exposure. Consequently, the present results were snapshots of memory retention over time after a simulation course. Some heterogeneity in the onset of decline in performance between 3 months and 1 year was reported in the literature for Basic Life Support (BLS) [31, 32] and Advanced Life Support [32–34]. Blumenfeld has studied the long-term memory of ATLS cognitive knowledge after training. Blumenfeld et al. showed a 20% loss of knowledge in half of the participants at 3 years with a need to recycle between 3 and 4 years [24]. The results of our study based on a performance evaluation using a valid and reproducible scale (TAPAS), suggest that simulation-based training requires reiteration every 2 years. They also suggest, if not to retake the PEP course at 4 years, at least to maintain acceptable performance in these specific pediatric skills. A future study should assess performance after the recycling of the PEP course. Other trainings are proposed on such a model. The European Resuscitation Council proposes revalidation every 5 years of several skills such as the European Pediatric Life Support or Newborn Life Support [16]. Furthermore, the Resuscitation Council Worldwide and the American Heart Association recommend retraining of health personnel every 2 years [17, 18]. Among the participants included in the present study (before applying non-inclusion and exclusion criteria), only 6.9% (18/262) had benefited from simulation training carried out in addition to the 6-month PEP course. Indeed, economic and organizational difficulties may render it hard to multiply total re-training [35]. Insofar as trainees do not retain the knowledge and fluidity required to manage a given rare procedure, they are more quickly able to get back up to speed if refresher training from time to time or just-in-time training is proposed [36]. A recent study on the rare use of TransVenous Pacing in Emergency Medicine acknowledged the futility of trying to keep the aforementioned rate skills fresh after initial training [37]. Two recent resuscitation studies showed that performance was maintained at 1 year after an initial session if there was a 6-month revision session [38, 39]. In addition, other studies have shown that regular upgrading of skills is required with repetition of time-spaced simulation sessions [29, 40, 41].

#### Secondary outcomes

A better and faster application of procedures could have been expected in the group that had just finished the training (G1) compared to the others. Surprisingly, the prior group (G1) took a longer time to complete management of the simulated case. This could be explained by the performance of more procedures and therefore by more comprehensive management. The time to perform skills, such as pulse checking and using BVM, was the shortest in the group that had finished the PEP course at least 4 years previously (G4) and the control group (C). On the other hand, less than 50% of the procedural steps requested in management of an unconscious infant were achieved by this group. This could be understood as the benefit of an oversimplification of the algorithm due to lack of knowledge in pediatric BLS.

Gastric tube insertion was the procedure failed the most by all groups. Of note, PEP course training engaged the learners to perform it within 2 min, aiming to reduce the risk of stomach distension during BVM ventilation with its intrinsic complications (impairment of ventilation and cardiac output) [2]. Although this dip in performance was less preponderant in the group that had just performed the PEP course (G1), it was barely achieved by more than 50% of this group. One hypothesis is that this procedure is easier to forget as itis the main departure from the equivalent adult scenario, which is what emergency residents and physicians are commonly exposed to, as suggested by the literature [2, 42–44]. Moreover, these results suggest that pediatric simulation should be increased in such courses.

The level of perceived stress during simulation was higher in the group that had just finished the PEP course (G1) corresponding to a better level of performance. Perception might influence performance during simulation [45]. An explanation for this result of higher perceived stress and performance in the same group, suggesting an adaptive stress response, could be given by Yerkes-Dodson’s law [46]. Numerous studies have reported that a certain level of stress can improve technical performance [47, 48]. In contrast, other studies have shown that the technical skills of the novices decreased when they were subjected to additional stress, while the skills of experts remained stable [4, 49]. The results of the present study did not show a correlation between stress and technical performance. We speculate that intervention on an infant implies a level of stress much higher than on an adult whatever the level of performance because of a low volume – high stakes situation. We might consider that the relationship between stress and performance is more complex and involves other factors that should be studied [50, 51]. Surprisingly, there was no correlation between performance and level of clinical experience. The hypothesis was that the most experienced participants would be the best performers. It is probable that the emergency residents and physicians at all levels of clinical experience applied the adult BLS algorithm to the infant by ignoring or having forgotten the recommendations specific to the pediatric population [52] due to a lack of clinical practice. Since simulation-based training in rare procedures significantly improves performance [21, 29, 30], our results suggested that all emergency residents and physicians, regardless of their level of clinical experience, could benefit from a specific pediatric simulation program to maintain optimal performance. We hypothesize that this is due to the fact that physicians’ pediatric emergency skills decrease despite increasing clinical experience, and not due to an defect in the assessment tool. This tool was used by the same raters to assess participants during the PEP course.

### External validity

This study showed decline in performance of management of a simulated pediatric emergency case among a population of emergency residents and physicians. We think it could be similar to any low volume – high stakes situation in emergency medicine, implying specific technical procedures. In this study we were interested in rare pediatric emergency procedures to study long-term memory. We could also have mentioned infrequent emergencies of adults [33, 34], or technical procedures in an operating room [30, 40] or intensive care unit [21]. Because recruitment of the sample cohort was done on a voluntary basis, the participants were perhaps more confident in their performance and/or more performing. This could have influenced the results of self-confidence perception.

### Limitations

This study had a number of limitations. Firstly, it was not a prospective cohort study but an instantaneous photography of skill retention in different groups. A power calculation was not carried out because participants of the Grande Aquitaine region and in French Guyana were contacted directly and each participant who agreed to participate and met inclusion criteria was enrolled. The small sample size of each group was to some extent due to the sorting into five groups, which was necessary in order to analyze performance at different lengths of delay. Trends observed for some secondary objectives without significance could have been due to this size effect. Another limitation was the imbalance between groups: G1 included 5 residents while the others had fewer or zero; physicians were more experienced in the control group. Usually this course was taken at the end of a residency. Consequently, residents were more numerous in G1, because simulation was evaluated within 6 months of the training. As the evaluation was done at a greater distance from the course, there were fewer residents in the other groups. Finally, performance was assessed with a validated scale, which was not the case for the survey used for trainee perception. Clinical exposure to pediatric emergencies may have been a more useful criteria than years of clinical experience in this survey, however, it was deemed too difficult to obtain this information. Regarding retention of pediatric skills in adult emergency physicians, we speculate that these participants were rarely exposed to pediatric life-threatening events. All participants confirmed in the survey that they rarely encountered these situations. Moreover, the pediatricians or emergency physicians who were working in a pediatric emergency department were excluded from the study to attenuate the risk of heterogeneity of performance when exposed to pediatric life-threatening events.

## Conclusion

This study evaluated the long-term retention of the technical performance of specific pediatric procedures among emergency residents and physicians by a specific simulation session at different times after completion of a pediatric university course. Results showed a decline in performance, irrespective of experience, at 6-months after training and total loss of benefit at 4 years after training. Based on performance evaluation using a valid and reproducible scale, this study tends to objectively demonstrate the need for re-training every 2 years and if not, to retake the course before 4 years after training to maintain acceptable performance. That said, it is not always easy to determine the level of performance for skills and knowledge that would be acceptable, in order to reach and maintain a high level of competence ensuring patient safety.

Future research should focus on the same outcomes in a prospective cohort study, and should analyze the process of re-training and reactivation of memory by repeated training in order to determine what would be the optimal repetition interval between simulation sessions most likely to blunt memory decline over time.

## Supplementary information

Additional file 1: The prerequisite training in pediatric emergency procedures.

Additional file 2: Team Average Performance Assessment Scale (TAPAS).

Additional file 3: Questionnaire.

## Data Availability

All data analyzed during this study are included in the manuscript and supplemental files. Materials described in the manuscript, including all relevant raw data, are freely available to any scientist wishing to use them for non-commercial purposes, without breaching participant confidentiality. For more details, please contact the corresponding author.

## Abbreviations

ATLS: Advanced Trauma Life Support
BLS: Basic Life Support
BVM: Bag-Valve-Mask
PEP: Pediatrics Emergency Procedures
SBE: Simulation-based education
TAPAS: Team Average Performance Assessment Scale
