# Supplementary material for: Long-term retention assessment after simulation-based-training of pediatric procedural skills among adult emergency physicians: a multicenter observational study
Source: BMC Med Educ. 2019 Sep 11;19:348. doi: 10.1186/s12909-019-1793-6 (PMC6739955; doi:10.1186/s12909-019-1793-6)
Supplement: Supplementary file 1 — The prerequisite training in pediatric emergency procedures. [file 12909_2019_1793_MOESM1_ESM.docx]

Appendix 1

1. The prerequisite training in pediatric emergency procedures:

The university course of Pediatrics Emergency Procedures (PEP) is a voluntary pediatrics emergency training program. This course was given by the same three teachers during the entire period. Simulations were delivered by the same group of supervisors throughout this period, using the same instructional strategies and materials. The contents of the PEP university course are modified every five years when the recommendations of the European Resuscitation Council recommendations have been modified. Consequently, all participants took a similar course between 2010 and 2015. The PEP course is a 1/3 didactics - 2/3 simulations three-week training spread out over 6 months (one week every two months), running twice a year. Its objectives are:

- To perform emergency procedures in various pre-hospital or hospital pediatric emergencies, using low and high-fidelity simulation on various models according to the age of the child.

- To follow the recommended algorithms for various types of pediatric emergency care (seriously ill child, severe trauma), tested by low and high-fidelity simulation settings.

- To develop teamwork in the management of various pediatric emergency situations. After a didactic approach to leadership, communication, and decision-making, teamwork practice was tested using high-fidelity simulation.

2. The testing scenario:

A scenario was drawn from among a series of three scenarios concerning ventilation, hemodynamic or neurological life-threatening events. The participants had to manage an unconscious three-month-old infant during a videotaped and timed low-fidelity simulation. They had an emergency pediatric bag (identical to the one used in the French Emergency Medical Services) including: a stethoscope, a towel roll, a suction device, oral airways of different sizes, a gastric tube with a suction syringe of 60mL, oxygen prongs, a high-concentration oxygen mask, bag mask ventilation (BMV) with masks of different sizes, an intubation set with different sizes of endotracheal tubes with or without cuff, a laryngoscope with blades of different sizes, a Magill forceps, and an intra-osseous Cook® needle with two syringes of 10 mL and one of 2 mL.
