# Supplementary material for: Long-term retention assessment after simulation-based-training of pediatric procedural skills among adult emergency physicians: a multicenter observational study
Source: BMC Med Educ. 2019 Sep 11;19:348. doi: 10.1186/s12909-019-1793-6 (PMC6739955; doi:10.1186/s12909-019-1793-6)
Supplement: Supplementary file 2 — Team Average Performance Assessment Scale (TAPAS). [file 12909_2019_1793_MOESM2_ESM.docx]

**Simulation:**

**Date:**

**Supervisor:**

**Scenario: unconscious three-month-old infant**

**Participants’ feeling (on 0-10 scales):**

| Self-confidence | Pre-simulation Stress | Stress during simulation | Dissatisfaction | Realism of the scenario |
| --- | --- | --- | --- | --- |
|  |  |  |  |  |

T0 =

Maximal possible score =

**A – AIRWAY □ □ 2 □ 1 □ 0** Verbal R, reactivity (conscience), groan **□ □ 2 □ 1 □ 0** Upright position 30° (establish^t^/maintaining) **□ □ 2 □ 1 □ 0** Neck position: neutral, sniffing, extension **□ □ 2 □ 1 □ 0** Jaw-thrust & chin-lift (± head-tilt) **□ □ 2 □ 1 □ 0** Mouth: secretions, vomiting, FB **□ □ 2 □ 1 □ 0** Suction of mouth ± nose **□ □ 2 □ 1 □ 0** Extraction of a visible FB (finger sweep) **□ □ 2 □ 1 □ 0** Oral airway (if unconscious)

T3 =

T2 =

T1 =

Maximal possible score =

**B – BREATHING □ □ 2 □ 1 □ 0** RR, work, volume (± abdomen), oxygenation **□ □ 2 □ 1 □ 0** BMV: 5 cycles of 3’’ then RR=12/25-30/RR=40-60  **□ □ 2 □ 1 □ 0** Gastric tube

T6 =

T4 =

Maximal possible score =

**C – CIRCULATION** **□ □ 2 □ 1 □ 0** HR, skin, pulses, preload (liver, EJV, conj), BP

T5 =
