# Supplementary material for: Long-term retention assessment after simulation-based-training of pediatric procedural skills among adult emergency physicians: a multicenter observational study
Source: BMC Med Educ. 2019 Sep 11;19:348. doi: 10.1186/s12909-019-1793-6 (PMC6739955; doi:10.1186/s12909-019-1793-6)
Supplement: Supplementary file 3 — Questionnaire. [file 12909_2019_1793_MOESM3_ESM.docx]

Appendix 3: Questionnaire

**Questionnaire**

□ Male □ Female Hospital............................

□ Resident □Emergency physician

Years of work-experience......................................................................

Pediatric emergency training in curriculum Yes / No year.......

Simulation-based-training during curriculum Yes/No Which one?....... Year.......
